# Ferrocene-Containing Pseudorotaxanes in Crystals: Aromatic Interactions with Hammett Correlation

**DOI:** 10.3390/molecules27051745

**Published:** 2022-03-07

**Authors:** Yuji Suzaki, Tomoko Abe, Asami Takei, Yugo Fukuchi, Take-aki Koizumi, Kohtaro Osakada, Masaki Horie

**Affiliations:** 1 Laboratory for Chemistry and Life Science, Tokyo Institute of Technology, 4259 Nagatsuta, Yokohama 226-8503, Japan; ysuzaki@gmail.com (Y.S.); timocop0912@gmail.com (T.A.); asami.tke@gmail.com (A.T.); taameiya_yugo@yahoo.co.jp (Y.F.); 2 Advanced Instrumental Analysis Center, Shizuoka Institute of Science and Technology, 2200-2 Toyosawa, Fukuroi 437-8555, Shizuoka, Japan; koizumi.takeaki@sist.ac.jp; 3 National Institute of Advanced Industrial Science and Technology (AIST), Tsukuba Central 5, 1-1-1 Higashi, Tsukuba 305-8565, Ibaraki, Japan; 4 Department of Chemical Engineering, National Tsing-Hua University, 101, Section 2, Kuang-Fu Road, Hsinchu 30013, Taiwan; mhorie@mx.nthu.edu.tw

**Keywords:** pseudorotaxane, aromatic interaction, crystal structure, Hammett constants

## Abstract

Single crystals of pseudorotaxanes, [(FcCH_2_NH_2_CH_2_Ar)(DB24C8)][PF_6_] (DB24C8 = dibenzo[24]crown-8, Fc = Fe(C_5_H_4_)(C_5_H_5_), Ar = -C_6_H_3_-3,4-Cl_2_, -C_6_H_3_-3,4-F_2_, -C_6_H_4_-4-F, -C_6_H_4_-4-Cl, -C_6_H_4_-4-Br, -C_6_H_3_-3-F-4-Me, -C_6_H_4_-4-I) and [(FcCH_2_NH_2_CH_2_C_6_H_4_-4-Me)(DB24C8)][Ni(dmit)_2_] (dmit = 1,3-dithiole-2,4,5-dithiolate), were obtained from solutions containing DB24C8 and ferrocenylmethyl(arylmethyl)ammonium. X-ray crystallographic analyses of the pseudorotaxanes revealed that the aryl ring of the axle moiety and the catechol ring of the macrocyclic component were at close centroid distances and parallel or tilted orientation. The structures with parallel aromatic rings showed correlation of the distances between the centroids to Hammett substituent constants of the aryl groups.

## 1. Introduction

Rotaxanes and pseudorotaxanes have been investigated as supramolecules with unique structures, having macrocyclic molecules threaded by axle molecules [1,2,3,4,5,6,7,8,9]. Their stimulus–response behavior has been applied to molecular shuttles [10,11,12,13,14,15,16,17,18] and molecular elevators [19] in solutions and molecular muscles in both the solid state and in solution [20,21,22,23]. Further applications of the rotaxanes include catalysis [24,25,26,27,28], functional polymeric materials [29,30,31,32], amphiphilic materials [33,34,35,36], and nanometal precursors [37]. Pseudorotaxanes have an axle component whose end groups are smaller than the size of the central hole of the macrocyclic component. Intermolecular interaction, such as hydrogen bonding and aromatic interaction, stabilizes the interlocked structure of the pseudorotaxanes. Dibenzo-24-crown-8 (DB24C8) forms various pseudorotaxanes and rotaxanes with dialkyl- or diarylammonium because they are bound by multiple N-H⋯O hydrogen bonds between the NH_2_^+^ group and the oxygen atoms. X-ray crystallographic studies of the pseudorotaxane of DB24C8 and dibenzylammonium, [((PhCH_2_)_2_NH_2_)(DB24C8)][PF_6_], revealed interaction between the macrocyclic and axle components [38,39]. The pseudorotaxane contained multiple N-H⋯O hydrogen bonds between the NH groups of the axle component and O atoms of the macrocycle. One of the two crystallographically independent pseudorotaxanes was stabilized not only by the hydrogen bonds but also by π–π interaction between a Ph group of the axle component and a catechol group of the macrocycle. The two aromatic groups had parallel orientation and close positions with a centroid distance of 3.79 Å. Such double stabilization was observed in many rotaxanes and pseudorotaxanes of DB24C8 and bis(arylmethyl)ammonium [40,41,42,43].

**Scheme 1:**
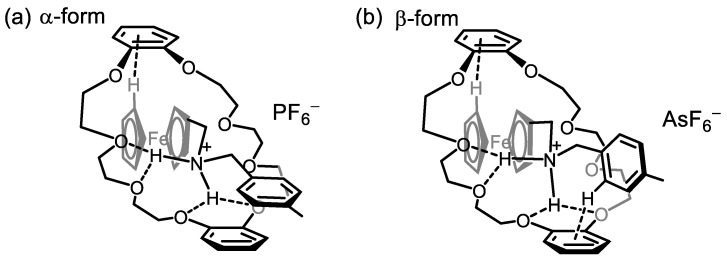
Typical structures of pseudorotaxane of DB24C8 and (ferrocenylmethyl)-(4-methylphenymethyl) ammonium.

In the last few decades, we have investigated structures and properties of the crystalline pseudorotaxanes of DB24C8 and ferrocenylmethyl(arylmethyl)ammonium, [(FcCH_2_NH_2_CH_2_C_6_H_4_-4-Me)(DB24C8)][EF_6_] (E = P, As) [44,45,46,47,48,49,50]. The pseudorotaxanes caused the crystalline phase transition upon heating and photo-irradiation. Related crystalline supramolecules were reported to exhibit new stimulus–response behavior [51,52,53,54]. Scheme 1 shows two structures of the pseudorotaxane of DB24C8 with ferrocenylmethyl(4-methylphenylmethyl)ammonium in the crystals. The pseudorotaxane with the PF_6_^−^ counter anion was supported by multiple N-H⋯O hydrogen bonds, π–π interaction between the 4-methylphenyl group and a catechol group, and C(Cp)–H⋯π interaction between the ferrocenyl group and the other catechol group (Scheme 1a, α–form). The distance and angle of the catechol ring and *p*-methylphenyl ring was determined to be 3.71 Å and 6.2°, respectively. The pseudorotaxane with AsF_6_^−^ anion preferred the structure with C⋯H–π interaction between the 4-methylphenyl group of the axle component and a catechol group (Scheme 1b, β–form). Heating crystals of [(FcCH_2_NH_2_CH_2_C_6_H_4_-4-Me)(DB24C8)]PF_6_ above 128 °C caused thermal crystalline phase transition from α–form to β–form. Recent studies revealed that the crystalline phase transition temperature of the crystals was influenced largely by size of the counter anions [47,50].

In solution, the pseudorotaxanes of DB24C8 and benzyl(arylmethyl)ammonium, [(ArCH_2_(PhCH_2_)NH_2_)(DB24C8)][PF_6_] have been reported to show different stabilities, depending on the substituents on the aromatic groups of the axle component [55]. However, there have been no reports on relevance of the crystalline structures of such (pseudo)rotaxanes to the substituents of the aromatic group of the arylmethylammonium axle component. Here we report the crystal structures of pseudorotaxanes composed of DB24C8 and ferrocenylmethyl(arylmethyl)ammonium and show the effect of the aryl group on the molecular structures of the pseudorotaxanes. This study focuses on relative positions and orientation of the neighbouring aromatic groups of the axle and cyclic components in the α–form pseudorotaxane crystals.

## 2. Results and Discussion

Mixing DB24C8 with ferrocenylmethyl(arylmethyl)ammonium in solution caused crystal growth of the corresponding pseudorotaxanes. Their structures were determined by X-ray crystallography (vide infra). The reaction of DB24C8 with ferrocenylmethyl(arylmethyl)ammonium formed the corresponding pseudorotaxanes, **1a**–**1g**, as crystals, as shown in Equation (1). Similar pseudorotaxanes with Ph, C_6_H_4_-4-Me, and C_6_H_4_-4-OMe groups in the axle component, **1h**–**1i**, were reported previously [46,47,48]. Counter anion exchange of [(FcCH_2_NH_2_CH_2_C_6_H_4_-4-Me)][PF_6_] by [Ni(dmit)_2_]^–^ (dmit = 1,3-dithiole-2-4,5-dithiolate) and subsequent addition of DB24C8 formed [(FcCH_2_NH_2_CH_2_C_6_H_4_-4-Me)(DB24C8)][Ni(dmit)_2_] (**1i-Ni**). X-ray crystallographic study showed the pseudorotaxane structure with [Ni(dmit)_2_] counter anion, although IR measurement and elemental analyses of the crystalline product were unsuccessful.


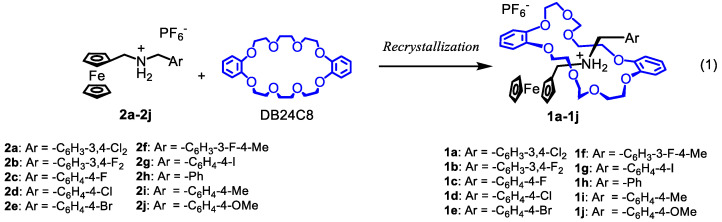


**Figure 1:**
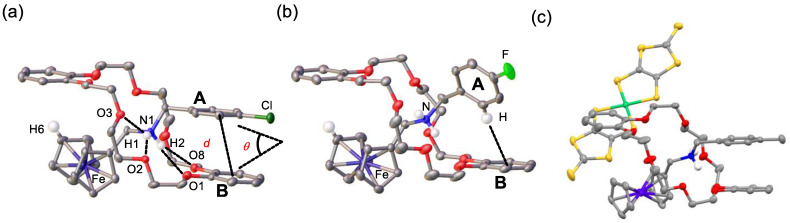
X-ray structures of pseudorotaxanes. (**a**) **1d**, (**b**) **1c**, and (**c**) **1i-Ni.** The atoms are shown with 50% probabilities. Atoms of the anion and hydrogen atoms are omitted for simplicity.

Figure 1a shows structure of pseudorotaxane **1d** with a chlorophenyl group in the axle component. The ammonium hydrogens, H1 and H2, are at close positions to the oxygen atoms of DB24C8 (N1–H1⋯O2: 2.216 Å, N1–H1⋯O3: 2.240 Å, N1–H2⋯O1: 2.537 Å, N1–H2⋯O8: 2.368 Å), suggesting N–H⋯O hydrogen bonds. The cyclopentadienyl ligand forms a C–H⋯π interaction (3.09 Å) with a C_6_H_4_ ring of DB24C8. The distance between the centroid of phenylene ring **A** of the axle component and that of the catechol ring **B** of DB24C8 (*d*, Å) and the angle formed by the aromatic planes (*θ*, °) are 3.70 Å and 5.26°, respectively. Thus, the structure of pseudorotaxane **1d** belongs to the α–form of Scheme 1.

**Figure 2:**
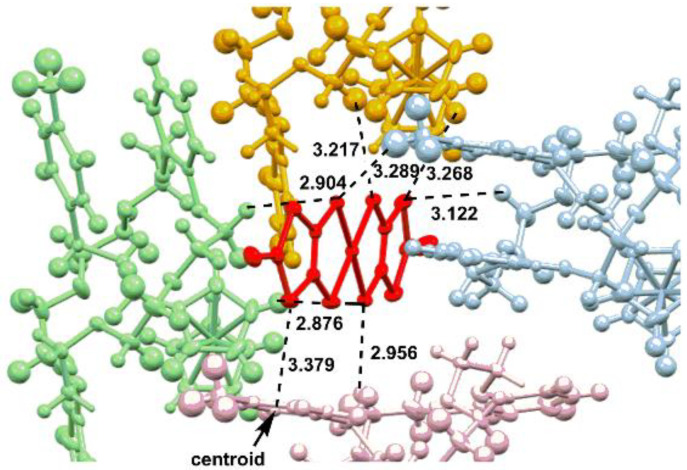
Contacts between the cationic rotaxanes and [Ni(dmit)_2_]^−^ of **1i-Ni**.

Pseudorotaxane **1c** has two aromatic groups **A** and **B** with the orientation close to orthogonal, suggesting C-H⋯π interaction between the aromatic groups **A** and **B**, as shown in Figure 1b. The structure is similar to **1i** above the crystalline phase transition temperature and belongs to β–form in Scheme 1. Pseudorotaxane **1i-Ni** contains the 4-methylphenyl group of the axle component and a phenylene group of a catechol group in parallel fashion, as shown in Figure 1c. Previous crystallographic studies of **1h**, **1i**, and **1j** showed that multiple C–H⋯F interactions between DB24C8 and PF_6_^−^ impart the relative stability of α– to the β–form [46,47,50]. Figure 2 depicts interaction of the cationic pseudorotaxane with [Ni(dmit)_2_]^−^ anion of **1i-Ni**, which differs largely from that of pseudorotaxane **1i** with PF_6_^−^ anions.

Table 1 summarizes synthesis and structures of the ferrocene-containing pseudorotaxanes, [(FcCH_2_NH_2_CH_2_Ar)(DB24C8)][PF_6_] (**1a**–**1j**) and [(FcCH_2_NH_2_CH_2_Ar)(DB24C8)] [Ni(dmit)_2_] (**1i-Ni**). The IR peaks of the symmetric and asymmetric vibration of ammonium N–H bonds of **1a**–**1d**, **1f**, **1g** (3065–3080, 3166–3195 cm^−1^) were observed at lower wavenumbers than those of starting ammonium, **2a**–**2g** (3233–3236 and 3262–3268 cm^−1^), due to the hydrogen bonding between the ammonium and oxygen atoms of DB24C8.

The pseudorotaxane crystals of **1i** in α–form and in β–form were reported to have different conformation of the axle molecule and co-conformation of the axle and macrocyclic molecules (orientation of the axle molecule within the pseudorotaxane framework) [50]. The rotaxanes in α–form (**1a**, **1d**–**1i**) and those in β–form (**1b**, **1c**, **1h**) in Table 1 showed different wavenumbers of the IR peaks due to ν_as_ vibrations of the NH_2_ group, 3184 cm^−1^ on average for **1a** and **1d**–**1i** and 3161 cm^−1^ on average for **1b**, **1c**, and **1h**. The distances between N1 and O2 atoms of **1a** and **1d**–**1i**, 3.079 Å on average, were longer than those of **1b**, **1c**, and **1h** (2.960 Å on average). Thus, these spectroscopic and structural parameters relating to the N–H⋯O hydrogen bonds differ clearly between the crystals of α–form and those of β–form. Table 2 summarizes relative positions and orientations of two aromatic planes **A** and **B** of the pseudorotaxanes in the crystalline state. The two aromatic planes of α–form were almost parallel in the structures with tilt angles, in the range of 3.65–7.94°. Distances between centroids of aromatic planes **A** and **B**, are in the range of 3.573–3.779 Å. Both values are much smaller than the corresponding values of pseudorotaxanes in β–form, **1b**, **1c**, and **1h**.

**Figure 3:**
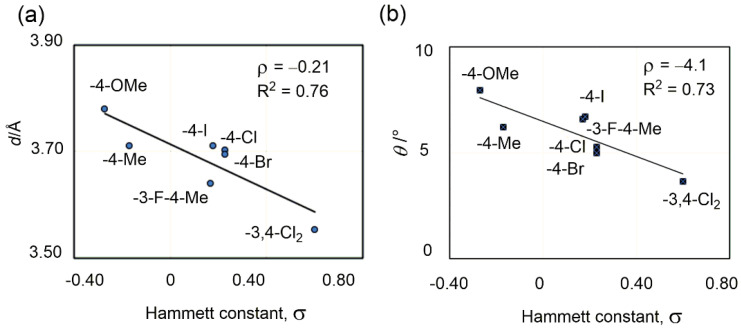
Hammett correlations of (**a**) *d* and (**b**) *θ* to σ. Hammett constants were taken from ref. [56] by assuming their additivity.

Figure 3 shows Hammett plots of structural parameters of crystalline pseudorotaxanes with α–form, **1a**, **1d**–**1g** and **1i**–**1j.** The distances between centroids (Figure 3a) and dihedral angles (Figure 3b) of aromatic groups **A** and **B** were plotted against the Hammett constants, σ, of **A** [56]. Hammett constants of disubstituted aromatic group in **1a** and **1f** were calculated by assuming additivity of Hammett constants [57,58]. Linear relationships were observed for *d* and *θ* values to Hammett constants, and σ-values were calculated as −0.21 and −4.1, respectively. Thus, aromatic group **A** with a larger σ-value was positioned at a closer position to aromatic group **B** with a smaller dihedral angle. Coefficients of determination of the plots in Figure 3a,b were similar (R^2^ = 0.76 and 0.73), suggesting that parameters *d* and *θ* were correlated with each other. Attempts to plot averaged distances between aromatic planes of **A** and **B** to Hammett constants resulted in lower correlation than that between *d* and *θ*. The two aromatic planes were almost parallel in the structures but had slight differences in the structural parameters. The *d* and *θ* values of pseudorotaxanes were increased by electron-donating substituents (negative σ values) of the terminal aryl group of the axle component.

Centroid distances of the pseudorotaxanes with monosubstituted aromatic groups **A**, such as **1d**, **1e**, **1g**, **1i**, and **1j**, were plotted against R^+^ constants in order to estimate the contribution of the resonance effect for the Hammett plots in Figure 3. Centroid distance of pseudorotaxane **1j** with OMe group at the 4-position of **A** (3.779 Å) is much longer than other pseudorotaxanes with Cl, Br, I, and Me groups. The coefficient of determination obtained from the plots of the five pseudorotaxanes is high (R^2^ = 0.95). This indicates that resonance effect of the aromatic group **A** is significant among the mono-substituted aromatic groups. These results indicate that the pseudorotaxanes bearing mono- and disubstituted aromatic group **A** showed that the electronic nature of **A** influenced the relative positions and orientations of the aryl groups **A** and **B**.

The centroid distance (*d*) and dihedral angle (*θ*) of **1i-Ni** (3.665 Å and 4.59°) were smaller than those of **1i** with PF_6_ anion (3.710 Å and 6.20°). Such effects of the counter anion on the structure of cationic pseudorotaxane are ascribed to the different co-conformation of the pseudorotaxanes caused by the counter anions (vide supra) [50].

**Scheme 2:**
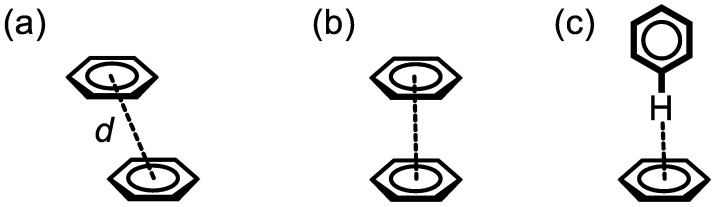
Typical geometries of benzene dimers, (**a**) slipped-parallel, (**b**) parallel, and (**c**) perpendicular.

Theoretical studies compared three possible geometries for the aromatic interactions, slipped-parallel, parallel, and perpendicular ones (Scheme 2). Tsuzuki et al. calculated stabilities of the benzene dimers as the function of distance (*d*) and angles (*θ*) between them and reported the optimized position for slipped paralleled conformation (*d* = 3.5 Å, Δ*G°* = −2.48 kcal mol^−1^ (at the CCSD(T) level)) which is more stable than the parallel type interaction (Δ*G°* = −1.48 kJ mol^−1^) and similar to C-H⋯π interaction (Δ*G°* = −2.46 kJ mol^−1^) [59,60,61,62]. Thus, the energy differences among the possible interacted structures are small. Recently, parallel stacking of the aromatic rings (Scheme 2b) was found in the crystals of polyhedral oligomeric silsesquioxane (POSS) derivatives, although it was considered to be less stable than the others [63]. The combination of two aromatic rings at close positions was known to influence stability of their π–π stacking. As a further important factor, donor–acceptor interaction was known to stabilize the aromatic interaction significantly.

**Figure 4:**
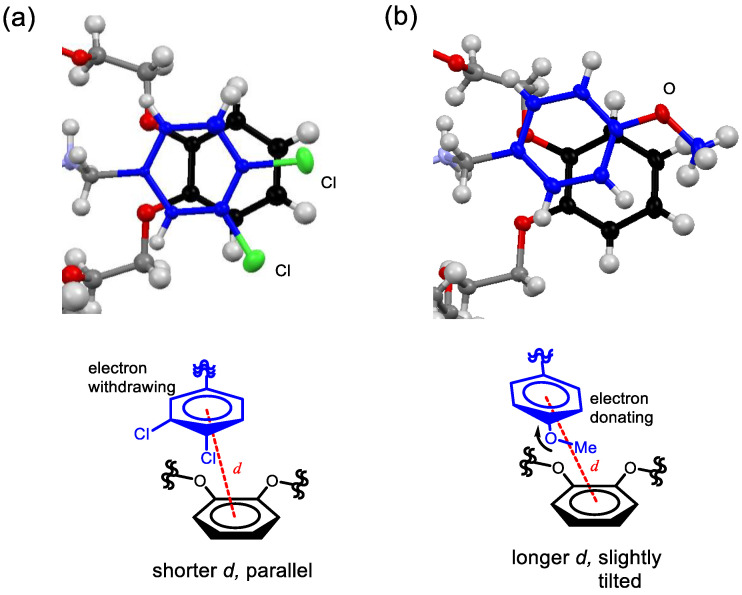
Partial structures of pseudorotaxanes (**a**) **1a** (left) and (**b**) **1j** (right). Two close aromatic rings by crystallographic results and their schematic diagram are shown.

Figure 4 depicts partial X-ray structures of **1a** and **1j**, showing the relative positions of their **A** and **B** rings. Both **A** and **B** rings of **1a** and **1j** show slipped-parallel type stacking (Scheme 2a). The overlapping of π electrons between **A** and **B** rings of **1a** looks larger than that of **1i**. The *d* and *θ* values of **1a** (3.553 Å and 3.65°) and of **1j** (3.779 Å and 7.94°) indicate that the C_6_H_3_-3,4-Cl_2_ ring of **1a** and the catechol ring of DB24C8 is closer and less tilted than those of the C_6_H_3_-4-OMe ring of **1i** and the catechol ring of DB24C8 because of stronger donor–acceptor interaction in the former system.

Stoddart, Williams, and their co-workers investigated a full series of pseudorotaxanes composed of DB24C8 and bis(arylmethyl)ammonium in the solid state and in solution. They observed a clear relationship between the stability constants for the pseudorotaxane and the electron donating ability of the substituents of the aryl groups of the axle components in CDCl_3_ and CD_3_CN-CDCl_3_ [55]. Higher stability of pseudorotaxanes possessing aryl groups with electron-withdrawing groups, such as NO_2_ and COOH groups, at the para position can be attributed to the aromatic interaction between the axle and macrocyclic components. Although direct estimation of the aromatic interaction was difficult in the solutions, the results in the solid of this study state are related to the relative stability of the pseudorotaxanes in the solution.

## 3. Materials and Methods

### 3.1. General

^1^H NMR spectra were acquired on a MERCURY300 (Varian, Tokyo, Japan), EX-400 (JEOL, Tokyo, Japan) and a AV-400M (Bruker, Yokohama, Japan). The chemical shifts were referenced with respect to CHCl_3_ (δ 7.26), CD_2_HCN (δ 1.93) for ^1^H, and CDCl_3_ (δ 77.0), CD_3_CN (δ 1.30) for ^13^C as internal standards. Elemental analysis was carried out with a CHNS-932 (LECO, Tokyo, Japan) or MT-5 CHN (Yanaco, Tokyo, Japan) autorecorder. IR spectra were measured with a FTIR-8100A (Shimadzu, Kyoto, Japan) and FT/IR-4100 (JASCO, Tokyo, Japan). H_2_NCH_2_Ar (Ar = -C_6_H_4_-4-Br, -C_6_H_3_-3-F-4-Me) was prepared by reaction of LiAlH_4_ and NCAr in THF under reflux condition. Other chemicals are commercially available and used without further purification.

### 3.2. Crystal Synthesis of [(FcCH_2_NH_2_CH_2_C_6_H_3_-3,4-Cl_2_)(DB24C8)]PF_6_ *(**1a**)*

Yellow crystals of pseudorotaxane **1a** were obtained by slow evaporation of CH_2_Cl_2_/Et_2_O solution of **2a** (52 mg, 0.10 mmol) and DB24C8 (46 mg, 0.10 mmol). **1a** was obtained with 10% yield. Synthesis details and spectroscopic results of the precursors of pseudorotaxanes and cif files are in Supplementary Materials.

Elemental analysis: calcd (%) for C_42_H_50_NO_8_FeF_6_Cl_2_P: C, 52.08; H, 5.20; N, 1.45; Cl, 7.32; found: C, 51.58; H, 4.93; N, 1.45; Cl, 7.38.

### 3.3. Crystal Synthesis of [(FcCH_2_NH_2_CH_2_C_6_H_3_-3,4-F_2_)(DB24C8)]PF_6_ *(**1b**)*

Yellow crystals of pseudorotaxane **1b** were obtained by slow evaporation of acetone/hexane solution of **2b** (46 mg, 0.093 mmol) and DB24C8 (49 mg, 0.11 mmol). **1b** was obtained with 57% yield.

Elemental analysis: calcd (%) for C_42_H_50_NO_8_FeF_8_P: C, 53.91; H, 5.39; N, 1.50; found: C, 53.81; H, 5.39; N, 1.52.

### 3.4. Crystal Synthesis of [(FcCH_2_NH_2_CH_2_C_6_H_4_-4-F)(DB24C8)]PF_6_ *(**1c**)*

Yellow crystals of pseudorotaxane **1c** were obtained by slow evaporation of acetone/hexane solution of **2c** (47 mg, 0.10 mmol) and DB24C8 (44 mg, 0.099 mmol). **1c** was obtained with 49% yield.

Elemental analysis: calcd (%) for C_42_H_51_NO_8_FeF_7_P: C, 54.97; H, 5.60; N, 1.53; found: C, 54.92; H, 5.65; N, 1.89.

### 3.5. Crystal Synthesis of [(FcCH_2_NH_2_CH_2_C_6_H_4_-4-Cl)(DB24C8)]PF_6_ *(**1d**)*

Yellow crystals of pseudorotaxane **1d** were obtained by slow evaporation of acetone/hexane solution of **2d** (48 mg, 0.10 mmol) and DB24C8 (45 mg, 0.10 mmol). **1d** was obtained with 74% yield.

Elemental analysis: calcd (%) for C_42_H_51_NClF_6_FeO_8_P: C, 54.00; H, 5.50; N, 1.50; found: C, 53.69; H, 5.45; N, 1.48.

### 3.6. Crystal Synthesis of [(FcCH_2_NH_2_CH_2_C_6_H_4_-4-Br)(DB24C8)]PF_6_ *(**1e**)*

Yellow crystals of pseudorotaxane **1e** were obtained by slow evaporation of CHCl_3_/acetone (2.0 mL/0.5 mL) solution of **2e** (53 mg, 0.10 mmol) and DB24C8 (45 mg, 0.10 mmol). **1e** was obtained with 85% yield.

Elemental analysis: calcd (%) for C_42_H_51_NBrF_6_FeO_8_P(H_2_O)_0.5_: C, 51.08; H, 5.31; N, 1.42; found: C, 50.99; H, 5.35; N, 1.45.

### 3.7. Crystal Synthesis of [(FcCH_2_NH_2_CH_2_C_6_H_3_-3-F-4-Me)(DB24C8)]PF_6_ *(**1f**)*

Yellow crystals of pseudorotaxane **1f** were obtained by slow evaporation of CH_2_Cl_2_/Et_2_O solution of **2f** (47 mg, 0.094 mmol) and DB24C8 (45 mg, 0.10 mmol). **1f** was obtained with 41% yield.

Elemental analysis: calcd (%) for C_43_H_53_NF_7_FeNO_8_P: C, 55.43; H, 5.73; N, 1.50; found: C, 55.45; H, 5.29; N, 1.55.

### 3.8. Crystal Synthesis of [(FcCH_2_NH_2_CH_2_C_6_H_4_-4-I)(DB24C8)]PF_6_ *(**1g**)*

Yellow crystals of pseudorotaxane **1g** were obtained by slow evaporation of CH_2_Cl_2_/Et_2_O solution of **2g** (57 mg, 0.10 mmol) and DB24C8 (45 mg, 0.10 mmol). **1g** was obtained with 69% yield.

Elemental analysis: calcd (%) for C_42_H_51_NIF_6_FeNO_8_P: C, 49.19; H, 5.01; N, 1.37; found: C, 49.29; H, 4.98; N, 1.37.

### 3.9. Crystal Synthesis of [(FcCH_2_NH_2_CH_2_C_6_H_4_-4-Me)(DB24C8)][Ni(dmit)_2_] *(**1i-Ni**)*

Compound [FcCH_2_NH_2_CH_2_C_6_H_4_-4-Me][Ni(dmit)_2_] (**2i-Ni**) was obtained by anion exchange of [FcCH_2_NH_2_CH_2_C_6_H_4_-4-Me][PF6] with Li[Ni(dmit)_2_]. Black crystals of pseudorotaxane **1i-Ni** were obtained by slow evaporation of CH_2_Cl_2_/Et_2_O solution of **2i-Ni** (77 mg, 0.10 mmol) and DB24C8 (45 mg, 0.10 mmol) with 43% yield. The crystals revealed the molecular structure but did not provide satisfactory analytical results.

### 3.10. X-ray Crystallography

Data were collected on a Rigaku Saturn CCD diffractometer with Mo Kα radiation (λ = 0.71073 Å). All H atoms were fixed at ideal positions. CCDC 1032569-1032577 and 2122024 contain the supplementary crystallographic data for complex **1a-1g**, and **1i-Ni**. Table 2 summarizes the structural data used in Figure 3a,b.

## 4. Conclusions

The pseudorotaxanes of DB24C8 and ferrocenylmethyl(arylmethyl)ammonium in this study were stabilized by three-point support in the crystals, N-H⋯O hydrogen bonds, C(Cp)–H⋯π interaction between the ferrocenyl group and a catechol group, and aromatic interaction between the aryl group of the axle component and a catechol group. Choice of the third interaction, either π⋯π interaction (α–form) or C-H⋯π interaction (β–form), was reported to depend on the counter anions, as shown for **2i** in our previous papers [46,47,50]. Crystals in α–form and those in β–form exhibit different IR peak positions and N⋯O distance, relating to the hydrogen bonds between the axle and macrocyclic molecules, as shown in Table 1. This study revealed that the aryl group of the axle molecules influenced structural parameters around the two aromatic groups with a π⋯π interaction. The aryl group with electron-withdrawing substituents enhanced the aromatic interaction and stabilized the pseudorotaxane. Findings in this study deepened full understanding of structures of the pseudorotaxanes with a ferrocenyl group.

## Figures and Tables

**Table 1:** Crystal synthesis of pseudorotaxanes, **1a**–**1j**.

Pseudorotaxane	End Group of Axle Component (Hammett Const.)		Yield(%)	Structure	N⋯O distance/Å		ν(N–H)/cm^−1 a^	
					Ν1–O2	Ν1–O3	ν_s_	ν_as_
**1a**	-C_6_H_3_-3,4-Cl_2_	(0.60)	10%	α	3.058	3.011	3060 (3233)	3185 (3262)
**1b**	-C_6_H_3_-3,4-F_2_	(0.40)	57%	β	2.975	2.840	3066 (3233)	3163 (3262)
**1c**	-C_6_H_4_-4-F	(0.34)	49%	β	2.966	2.873	3077 (3236)	3165 (3262)
**1d**	-C_6_H_4_-4-Cl	(0.23)	74%	α	3.088	2.949	3069 (3235)	3187 (3266)
**1e**	-C_6_H_4_-4-Br	(0.23)	85%	α	3.092	2.943	3069 (3233)	3187 (3265)
**1f**	-C_6_H_3_-3-F-4-Me	(0.17)	41%	α	3.062	3.006	3067 (3233)	3166 (3266)
**1g**	-C_6_H_4_-4-I	(0.18)	69%	α	3.070	2.984	3065 (3235)	3195 (3268)
**1h** ^b^	-Ph	(0.00)		β	2.941	2.857	3065 (3236)	3156 (3262)
**1i** ^c^	-C_6_H_4_-4-Me	(−0.17)		α	3.159	3.074	3067 (3250)	3171 (3225)
**1i-Ni**	-C_6_H_4_-4-Me	(−0.17)		α	3.114	3.021	−	−
**1j** ^d^	-C_6_H_4_-4-OMe	(−0.27)		α	2.995	2.997	3065	3195

^a^ Wavenumber of the axle molecule is shown in parenthesis. ^b^ Ref. [47]. ^c^ Refs. [44,45]. ^d^ Ref. [55].

**Table 2:** Centroid distance and dihedral angles between the two aromatic groups in the axle and cyclic components.

Pseudorotaxane A Ring		Structure	Relative Positions of Two Aromatic Groups		
			Centroid Distance, *d*/Å	Dihedral Angle, *θ*/°	Face-to-Face Dimension/Å
**1a**	-C_6_H_3_-3,4-Cl_2_	α	3.553	3.65	3.376
**1b**	-C_6_H_3_-3,4-F_2_	β	4.850	122.01	–
**1c**	-C_6_H_4_-4-F	β	5.140	121.78	–
**1d**	-C_6_H_4_-4-Cl	α	3.702	5.26	3.416
**1e**	-C_6_H_4_-4-Br	α	3.694	5.00	3.394
**1f**	-C_6_H_3_-3-F-4-Me	α	3.640	6.59	3.413
**1g**	-C_6_H_4_-4-I	α	3.710	6.71	3.435
**1h** ^a^	-Ph	β	5.128	57.61	–
**1i** ^a^	-C_6_H_4_-4-Me	α	3.710	6.20	3.430
**1i-Ni**	-C_6_H_4_-4-Me	α	3.665	4.59	3.398
**1j** ^a^	-C_6_H_4_-4-OMe	α	3.779	7.94	3.529

^a^ Data were taken from the results in refs. [44,45,46,47,50].

## Data Availability

Experimental details are obtained from Supporting Information. CCDC 1032570-10325575 and 2122024 contain the supplementary crystallographic data for this paper. These data can be obtained free of charge via www.ccdc.cam.ac.uk/data_request/cif,orby (accessed on 15 December 2021), emailing data_request@ccdc.cam.ac.uk, or by contacting The Cambridge Crystallographic Data Centre, 12 Union Road, Cambridge CB2 1EZ, UK; Fax: +44-1223-336033.

## Supplementary Materials

The following supporting information can be downloaded online, synthesis details and spectroscopic results of the precursors of pseudorotaxanes and cif files of **1b–1g** and **1i-Ni**.

## Sample Availability

No samples of the compounds are available from the authors.
