# Supplementary material for: Ferrocene-Containing Pseudorotaxanes in Crystals: Aromatic Interactions with Hammett Correlation
Source: Molecules. 2022 Mar 7;27(5):1745. doi: 10.3390/molecules27051745 (PMC8911870; doi:10.3390/molecules27051745)
Supplement: Supplementary file 1 [file molecules-27-01745-s001.zip › molecules-1555769-supplementary.pdf]

Supporting Information

Ferrocene-containing Pseudorotaxanes in Crystals: Aromatic Interactions with Hammett Correlation

Yuji Suzaki,[1] Tomoko Abe,[1] Asami Takei, [1] Yugo Fukuchi, [1] Take-aki Koizumi,[2] Kohtaro Osakada[1,3]* and Masaki Horie[4]

[1] Laboratory for Chemistry and Life Science, Tokyo Institute of Technology, 4259 Nagatsuta, Yokohama 226-8503, Japan.

[2] Advanced Instrumental Analysis Center, Shizuoka Institute of Science and Technology, 2200-2 Toyosawa, Fukuroi, Shizuoka 437-8555, Japan.

[3] National Institute of Advanced Industrial Science and Technology (AIST), Tsukuba Central 5, 1-1-1 Higashi, Tsukuba, Ibaraki 305-8565, Japan

[4] Department of Chemical Engineering, National Tsing-Hua University, 101, Section 2, Kuang-Fu Road, Hsinchu 30013, Taiwan

* Correspondence: kosakada@res.titech.ac.jp; Tel.: +81-45-924-5224 (K.O.)

Experimental section 1
Table S1. IR and T_g data. 7
Table S2. Crystallographic results 8

Experimental section

General

^{1}H and ^{13}C{^{1}H} NMR spectra were acquired on a MERCURY300 (Varian), EX-400 (JEOL) and a AV-400M (Bruker). The chemical sifts were referenced with respect to $CHCl_3$ (δ 7.26), CD_2HCN (δ 1.93) for ^{1}H, and $CDCl_3$ (δ 77.0), CD_3CN (δ 1.30) for ^{13}C as internal standards. Elemental analysis was carried out with a CHNS-932 (LECO) or MT-5 CHN (Yanaco) autorecorder. IR spectra were measured with a FTIR-8100A (Shimadzu) and FT/IR-4100 (JASCO). DSC was recorded by Seiko instrument DSC6200S. Thermogravimetric analysis (TGA) was recorded on Seiko TG/DTA6200R. H_2NCH_2Ar (Ar = $-C_6H_4$-4-Br, $-C_6H_3$-3-F-4-Me) was prepared by reaction of $LiAlH_4$ and NCAr in THF under reflux condition. Other chemicals were commercially available and used without further purification.

Synthesis of $FcCH{=}NCH_2C_6H_4$*-3,4-*Cl_2 ***(3a)*** *(Fc = Fe(*C_5H_4*)(*C_5H_5*))*

A solution of ferrocenecarboxaldehyde (1.07 g, 5.0 mmol) and $H_2NCH_2C_6H_4$-3,4-Cl_2 (0.89 g, 5.1 mmol) in EtOH (100 mL) was stirred for 14 h at 65 °C in the presence of MS4A (molecular sieves 4A, 1.0 g). The reaction mixture was filtered and then evaporated to yield the crude product which was purified by washing with hexane to yield $FcCH{=}NCH_2C_6H_4$-3,4-Cl_2 as a reddish-brown solid (1.69 g, 4.5 mmol, 90%). ^{1}H NMR (300 MHz, $CDCl_3$, r.t.): δ = 4.19 (s, 5H, C_5H_5), 4.41 (m, 2H, C_5H_4), 4.60 (s, 2H, NCH_2), 4.66 (m, 2H, C_5H_4), 7.15 (d, 1H, C_6H_3), 7.39 (d, 1H, C_6H_3), 7.42 (d, 1H, C_6H_3), 8.24 (s, 1H, N=CH); ^{13}C{^{1}H} NMR (100 MHz, $CDCl_3$, r.t.): δ = 63.6 (NCH_2), 68.4 (C_5H_5), 69.1 (C_5H_4), 70.6 (C_5H_4), 79.8 (C_5H_4), 126.9 (C_6H_3), 129.4 (C_6H_3), 130.2, (C_6H_3), 130.5 (C_6H_3), 132.1 (C_6H_3), 140.0 (C_6H_3), 163.2 (N=CH); IR (KBr disk): ν(C=N) = 1636 cm^{-1}; elemental analysis: calcd (%) for $C_{18}H_{15}NCl_2Fe(H_2O)_{0.5}$: C 56.73, H 4.23, N 3.68; found: C 56.91, H 3.98, N 3.70.

Synthesis of $FcCH{=}NCH_2C_6H_3$*-3,4-*F_2 ***(3b)***

Compound **3b** was prepared in the same way as **3a**. The reaction of ferrocene-carboxaldehyde (1.07 g, 5.0 mmol) and H_2NCH_2-C_6H_3-3,4-F_2 (0.71 g, 5.0 mmol) in EtOH (100 mL) for 20 h at 65 °C in the presence of MS4A (1.0 g) yielded **3b** (1.66 g, 4.9 mmol, 98%) as a reddish-brown solid. ^{1}H NMR (300 MHz, $CDCl_3$, r.t.): δ = 4.19 (s, 5H, C_5H_5), 4.40 (s, 2H, NCH_2), 4.59 (m, 2H, C_5H_4), 4.68 (m, 2H, C_5H_4), 6.90–7.19 (3H, C_6H_3), 8.24 (s, 1H, N=CH); IR (KBr disk): *ν*(C=N) = 1638 cm^{-1}; elemental analysis: calcd (%) for $C_{18}H_{15}NF_2Fe(H_2O)_{0.25}$, C 62.91, H 4.55, N 4.08, F 11.06; found: C 63.23, H 4.26, N 4.04, F 11.27.

*Synthesis of $FcCH{=}NCH_2C_6H_4$-4-F (**3c**)*

Compound **3c** was prepared in the same way as **3a**. The reaction of ferrocenecarboxaldehyde (1.07 g, 5.0 mmol) and H_2 $NCH_2C_6H_4$-4-F (626 mg, 5.0 mmol) in toluene (100 mL) for 12 h at 75 °C in the presence of MS4A (1.0 g) yielded **3c** (1.29 g, 4.0 mmol, 80%) as a reddish-brown solid. ^{1}H NMR (300 MHz, $CDCl_3$, r.t.): δ = 4.17 (s, 5H, C_5H_5), 4.39 (m, 2H, C_5H_4), 4.68 (s, 2H, NCH_2), 4.62 (m, 2H, C_5H_4), 7.03 (d, 2H, C_6H_4, *J* = 8.7 Hz), 7.26 (d, 2H, C_6H_4, *J* = 8.7 Hz), 8.24 (s, 1H, N=CH); $^{13}C\{^1H\}$ NMR (75.5 MHz, $CDCl_3$, r.t.): δ = 64.3 (NCH_2), 68.5 (C_5H_4), 69.0 (C_5H_5), 70.5 (C_5H_4), 80.2 (C_5H_4), 115.2 (C_6H_4, *J*(FH) = 22 Hz), 129.3 (C_5H_4, *J*(FH) = 8 Hz), 135.3 (C_6H_4), 161.8 (C_6H_4, *J*(FH) = 245 Hz), 162.3 (N=CH); IR (KBr disk): *ν*(C=N) = 1638 cm^{-1}; elemental analysis: calcd (%) for $C_{18}H_{16}FFeN$: C 67.31, H 5.02, N 4.36; found: C 67.25, H 4.83, N 4.49.

*Synthesis of $FcCH{=}NCH_2C_6H_4$-4-Cl (**3d**)*

Compound **3d** was prepared in the same way as **3a**. The reaction of ferrocenecarboxaldehyde (2.14 g, 10 mmol) and $H_2NCH_2C_6H_4$-4-Cl (1.48 g, 10 mmol) in EtOH (200 mL) for 6 h at 65 °C in the presence of MS4A (2.0 g) yielded **3d** (2.80 g, 8.3 mmol, 80%) as a reddish-brown solid. ^{1}H NMR (300 MHz, $CDCl_3$, r.t.): δ = 4.18 (s, 5H, C_5H_5), 4.39 (m, 2H, C_5H_4), 4.62 (s, 2H, NCH_2), 4.68 (m, 2H, C_5H_4), 7.24 (d, 2H, C_6H_4, *J* = 8.7 Hz), 7.31 (d, 2H, C_6H_4, *J* = 8.7 Hz), 8.24 (s, 1H, NCH); $^{13}C\{^1H\}$ NMR (75.5 MHz, $CDCl_3$, r.t.): δ = 64.2 (NCH_2), 68.4 (C_5H_4), 68.9 (C_5H_5), 70.5 (C_5H_4), 80.1 (C_5H_4), 128.4 (C_6H_4), 129.0 (C_6H_4), 132.4 (C_6H_4), 138.1 (C_6H_4), 162.4 (NCH); IR (KBr disk): *ν*(C=N) = 1636 cm^{-1}; elemental analysis: calcd (%) for $C_{18}H_{16}ClFeN$: C 64.03, H 4.78, N 4.15; found: C 63.73, H 4.84, N 4.10.

*Synthesis of $FcCH{=}NCH_2C_6H_4$-4-Br (**3e**)*

Compound **3e** was prepared in the same way as **3a**. The reaction of ferrocene-carboxaldehyde (1.08 g, 5.0 mmol) and $H_2NCH_2C_6H_4$-4-Cl (939 mg, 5.0 mmol) in EtOH (80 mL) for 12 h at 65 °C in the presence of MS4A (1.0 g) yields **3e** (1.50 g, 3.9 mmol, 78%) as a reddish-brown solid. ^{1}H NMR (300 MHz, $CDCl_3$, r.t.): δ = 4.18 (s, 5H, C_5H_5), 4.39 (m, 2H, C_5H_4), 4.60 (s, 2H, NCH_2), 4.68 (m, 2H, C_5H_4), 7.18 (d, 2H, C_6H_4, *J* = 8.4 Hz), 7.46 (d, 2H, C_6H_4, *J* = 8.4 Hz), 8.23 (s, 1H, N=CH); $^{13}C\{^1H\}$ NMR (75.5 MHz, $CDC1_3$, r.t.): δ = 64.2 (NCH_2), 68.5 (C_5H_4), 69.0 (C_5H_5), 70.5 (C_5H_4), 80.0 (C_5H_4), 120.6 (C_6H_4), 129.4 ($C6_5H_4$), 131.4 (C_6H_4), 138.5 (C_6H_4), 162.6 (N=CH); IR (KBr disk): *ν* (C=N) 1636 cm^{-1}; elemental analysis: calcd (%) for $C_{18}H_{16}BrFeN(H_2O)_{0.25}$: C 55.92, H

4.30, N 3.62; found: C 55.98, H 4.12, N, 3.36.

*Synthesis of FcCH=NCH$_2$C$_6$H$_3$-3-F-4-Me (**3f**)*

Compound **3f** was prepared in the same way as **3a**. The reaction of ferrocenecarboxaldehyde (0.53 g, 2.5 mmol) and H$_2$NCH$_2$C$_6$H$_3$-3-F-4-Me (0.35 g, 2.5 mmol) in EtOH (100 mL) for 12 h at 65 °C in the presence of MS4A (1.0 g) yielded **3f** (0.76 g, 2.3 mmol, 92%) as a reddish-brown solid. ^{1}H NMR (300 MHz, CDC1$_3$, r.t.): δ = 2.54 (Me), 4.46 (s, 5H, C$_5$H$_5$), 4.67 (m, 2H, C$_5$H$_4$), 4.89 (s, 2H, NCH$_2$), 4.96 (ITI, 2H, C$_5$H$_4$), 7.24–7.54 (C$_6$H$_3$, 3H), 8.51 (s, 1H,N=CH); ^{13}C{^{1}H} NMR (75.5 MHz, CDC1$_3$, r.t.): δ = 14.2 (Me), 64.2 (NCH2), 68.6(C$_5$H$_4$), 69.0 (C$_5$H$_5$), 70.5 (C$_5$H$_4$), 80.3 (C$_5$H$_4$), 114.1 (C$_5$H$_3$ *J* (FC) = 22 Hz), 123.0 (C$_6$H$_3$ *J*(FC) = 16 Hz), 131.3 (C$_6$H$_3$, *J*(FC) = 5 Hz), 139.3 (C$_6$H$_3$), 161.1 (C$_6$H$_3$), 162.4 (N=CH); IR (KBr disk): ν(C=N) = 1634 cm^{-1}.

*Synthesis of FcCH=NCH$_2$C$_6$H$_4$-4-I (**3g**)*

Compound **3g** was prepared in the same way as **3a**. The reaction of ferrocenecarboxaldehyde (420 mg, 2.0 mmol) and H$_2$NCH$_2$C$_6$H$_4$-4-1 (477 mg, 2.0 mmol) in EtOH (20 mL) for 5 h at 65 °C in the presence of MS4A (1.0 g) yielded **3g** (711 mg, 1.7 mmol, 85%) as a reddish-brown solid. ^{1}H NMR (300 MHz, CDC1$_3$, r.t.): δ = 4.18 (s, 5H, C$_5$H$_5$), 4.39 (m, 2H, C$_6$H$_4$), 4.59 (s, 2H, NCH$_2$), 4.67 (m, 2H, C$_5$H$_4$), 7.06 (d, C$_6$H$_4$, *J* = 8.1 Hz), 7.66 (d, C$_6$H$_4$, *J* = 8.1 Hz), 8.23 (s, 1H, N=CH); ^{13}C{^{1}H}NMR (75.5 MHz, CDC1$_3$, r.t.): δ = 64.2 (NCH$_2$), 68.4 (C$_5$H$_4$), 68.9 (C$_5$H$_5$), 70.4 (C$_5$H$_4$), 80.0 (C$_5$H$_4$), 92.0 (C$_6$H$_4$), 129.6 (C$_6$H$_4$), 137.2 (C$_6$H$_4$),139.2 (C$_6$H$_4$), 162.4 (N=CH); IR (KBr disk): ν(C=N) = 1632 cm^{-1}; elemental analysis: calcd (%) for C$_{18}$H$_{16}$IFeN(H$_2$O)$_{0.25}$ C 49.86, H 3.84, N 3.23, I 29.27; found: C 49.82, H 4.08, N 3.26, I 28.92.

*Synthesis of [FcCH$_2$NH$_2$CH$_2$C$_6$H$_3$-3,4-Cl$_2$] PF$_6$ (**2a**)*

FcCH = NCH$_2$C$_6$H$_3$-3,4-Cl$_2$ (**3a**) (1.4 g, 3.8 mmol) was dissolved in MeOH (50 mL) at room temperature. NaBH$_4$ (1.55 g, 41 mmol) was added to the solution and it was stirred for 12 h at room temperature and then mixed with 4 M HCl (150 mL) to cause the separation of a solid from the solution. The resulting solid was filtered and washed with water and Et$_2$O to yield [FcCH$_2$NH$_2$CH$_2$C$_6$H$_3$-3,4-Cl$_2$]Cl which was used without further purification. Acetone (100 mL) suspension of [FcCH$_2$NH$_2$CH$_2$C$_6$H$_3$-3,4-Cl$_2$]Cl and NH$_4$PF$_6$ (3.26 g, 20 mmol) was stirred for 2 h at room temperature. The resulting mixture was filtered and the solids were washed with water, CH$_2$Cl$_2$ and hexane to give [FcCH$_2$NH$_2$CH$_2$C$_6$H$_3$-3,4-C1$_2$](PF$_6$) (**2a**) as a yellow solid (quant). ^{1}H NMR (300 MHz, CD$_3$CN, r.t.): δ = 4.03 (s, 2H, NCH$_2$), 4.07 (s, 2H, NCH$_2$), 4.20 (s, 5H, C$_5$H$_5$), 4.27

(m, 2H, C_5H_4), 4.36 (m, 2H, C_5H_4), 7.06 (s, 1H, C_6H_3), 7.34 (dd, 1H, C_6H_3, J = 8.5, 2.2 Hz), 7.59 (d, 1H, C_6H_3, J = 8.0 Hz); $^{13}C\{^1H\}$ NMR (75.5 MHz, CD_3CN, r.t.): δ = 48.5 (NCH_2), 50.0 (NCH_2), 69.9 (C_5H_4), 70.5 (C_5H_5), 71.4 (C_5H_4), 76.5 (C_5H_4), 131.0 (C_6H_3), 132.0 (C_6H_3), 132.6 (C_6H_3), 133.0 (C_6H_3), 133.2 (C_6H_3), 134.0 (C_6H_3); elemental analysis: calcd (%) for $C_{18}H_{18}NFeF_6Cl_2P$: C 41.57, H 3.49, N 2.69, Cl 13.63; found: C 41.39, H 3.38, N 2.72, Cl 13.88.

*Synthesis of [FcCH₂NH₂CH₂C₆H₃-3,4-F](PF₆) (**2b**)*

Compound **2b** was prepared in the same way as **2a** (82% from **3b**). ^1H NMR (300 MHZ, CD_3CN, r.t.): δ = 4.03 (s, 2H, NCH_2), 4.07 (s, 2H, NCH_2), 4.21 (s, 5H, C_5H_5), 4.28 (m, 2H, C_5H_4), 4.36 (m, 2H, C_5H_4), 7.24–7.38 (3H, C_6H_3); $^{13}C\{^1H\}$NMR (75.5 MHz, CD_3CN, r.t.): δ = 48.4 (NCH_2), 50.2 (NCH_2), 69.9 (C_5H_4), 70.5 (C_5H_5), 71.4 (C_5H_4), 76.6 (C_5H_4), 118.8 (C_6H_3), 120.1 (C_6H_3, J = 14, 5 Hz), 128.0 (C_6H_3, J(CF) = 7, 4 Hz), 129.2 (C_6H_3, J(CF) = 5, 5 Hz), 150.0 (C_6H_3, J(CF) = 73, 15 Hz), 152.4 (C_6H_3, J(CF) = 74, 14 Hz); elemental analysis: calcd (%) for $C_{18}H_{19}ClF_6FeNP$: C 44.38, H 3.72, N 2.88; found: C 44.35, H 3.76, N 2.93.

*Synthesis of [FcCH₂NH₂CH₂C₆H₄-4-F](PF₆) (**2c**)*

Compound **2c** was prepared in the same way as **2a** (47% from **3c**). ^1H NMR (300 MHz, CD_3CN, r.t.): δ = 4.04 (s, 2H, NCH_2), 4.09 (s, 2H, NCH_2), 4.21 (s, 5H, C_5H_5), 4.27 (m, 2H, C_5H_4), 4.37 (m, 2H, C_5H_4), 7.18 (d, 2H, C_6H_4, J = 8.8 Hz), 7.45 (d, 2H, C_6H_4, J = 8.8 Hz); $^{13}C\{^1H\}$ NMR (75.5 MHz, CD_3CN, r.t.): δ = 48.7 (NCH_2), 50.9 (NCH_2), 69.6 (C_5H_5), 70.5 (C_5H_4), 71.4 (C_5H_4), 76.4 (C_5H_4), 116.9 (C_6H_4, J(FC) = 22 Hz), 127.8 (C_6H_4, J(FC) = 3 Hz), 133.5 (C_6H_4, J(FC) = 9 Hz), 164.2 (C_6H_4, J(FC) = 247 Hz); elemental analysis: calcd (%) for $C_{18}H_{19}F_7FeNP$: C 46.08, H 4.08, N 2.99; found: C 46.37, H 3.93, N 3.07.

*Synthesis of [FcCH₂NH₂CH₂C₆H₄-4-Cl](PF₆) (**2d**)*

Compound **2d** was prepared in the same way as **2a** (65% from **3d**). ^1H NMR (300 MHz, CD_3CN, r.t.): δ = 4.05 (s, 2H, NCH_2), 4.09 (s, 2H, NCH_2), 4.21(s, 5H, C_5H_5), 4.28 (m, 2H, C_5H_4), 4.37 (m, 2H, C_5H_4), 7.40 (d, 2H, C_6H_4, J = 8.7 Hz), 7.46 (d, 2H, C_6H_4, J = 8.7 Hz); $^{13}C\{^1H\}$ NMR (75.5 MHz, CD_3CN, r.t.): δ = 48.2 (NCH_2), 50.4 (NCH_2), 69.6 (C_5H_5), 70.2 (C_5H_4), 71.1 (C_5H_4), 76.1 (C_5H_4), 129.7 (C_6H_4), 130.2 (C_6H_4), 132.4 (C_6H_4), 135.6 (C_6H_4); elemental analysis: calcd (%) for $C_{18}H_{19}ClF_6FeNP$: C 44.52, H 3.94, N 2.88; found: C 44.71, H 4.21, N 2.90.

*Synthesis of [FcCH$_2$NH$_2$CH$_2$C$_6$H$_4$-4-Br](PF$_6$) (**2e**)*

Compound **2e** was prepared in the same way as **2a** (38% from **3e**). ^{1}H NMR (300 MHz, CD$_3$CN, r.t.): δ = 4.04 (s, 2H, NCH$_2$), 4.07 (s, 2H, NCH$_2$), 4.21 (s, 5H, C$_5$H$_5$), 4.27 (m, 2H, C$_5$H$_4$), 4.37 (m, 2H, C$_5$H$_4$), 7.34 (d, 2H, C$_6$H$_4$, *J* = 8.4 Hz), 7.61 (d, 2H, C$_6$H$_4$, *J* = 8.4 Hz); ^{13}C{^{1}H} NMR (75.5 MHz, CD$_3$CN, r.t.): δ = 48.8 (NCH$_2$), 51.0 (NCH$_2$), 69.9 (C$_5$H$_5$), 70.5 (C$_5$H$_4$), 71.5 (C$_5$H$_4$), 76.2 (C$_5$H$_4$), 124.3 (C$_6$H$_4$), 130.8 (C$_6$H$_4$), 133.0 (C$_6$H$_4$), 133.1 (C$_6$H$_4$); elemental analysis: calcd (%) for C$_{18}$H$_{19}$BrF$_6$FeNP: C 40.79, H 3.61, N 2.64; found: C 40.43, H 3.58, N, 2.67.

*Synthesis of [FcCH$_2$NH$_2$CH$_2$C$_6$H$_3$-3-F-4-Me](PF$_6$) (**2f**)*

Compound **2f** was prepared in the same way as **2a** (59% from **3f**). ^{1}H NMR (300 MHz, CD$_3$CN, r.t.): δ = 2.27 (Me), 4.06 (s, 2H, NCH$_2$), 4.07 (s, 2H, NCH$_2$), 4.21 (s, 5H, C$_5$H$_5$), 4.28 (m, 2H, C$_5$H$_4$), 4.37 (m, 2H, C$_5$H$_4$), 7.11-7.14 (2H, C$_6$H$_3$), 7.30 (1H, C$_6$H$_3$); ^{13}C{^{1}H} NMR (75.5 MHz, CD$_3$CN, r.t.): δ = 48.9 (NCH$_2$), 51.0 (NCH$_2$), 70.1 (C$_5$H$_5$), 71.5 (C$_5$H$_4$), 76.3 (C$_5$H$_4$), 117.4 (C$_6$H$_3$, *J*(FC) = 23 Hz), 126.8 (C$_6$H$_3$, *J*(FC) = 4 Hz), 127.6 (C$_6$H$_3$, *J*(FC) = 17 Hz), 131.1 (C$_6$H$_3$, *J* (FC) = 8 Hz), 133.2 (C$_6$H$_3$, *J* (FC) = 5 Hz), 162.0 (C$_6$H$_3$, *J* (FC) = 254 Hz).

*Synthesis of [FcCH$_2$NH$_2$CH$_2$C$_6$H$_4$-4-I]PF$_6$ (**2g**)*

Compound **2g** was prepared in the same way as **2a** (50% from **3g**). ^{1}H NMR (300 MHz, CD3CN, r.t.): δ = 4.03 (s, 2H, NCH$_2$), 4.05 (s, 2H, NCH$_2$), 4.20 (s, 5H, C$_5$H$_5$), 4.27 (m, 2H, C$_5$H$_4$), 4.36 (m, 2H, C$_5$H$_4$), 7.19 (d, 2H, C$_6$H$_4$, *J* = 8.7 Hz), 7.81 (d, 2H, C$_6$H$_4$, *J* = 8.7 Hz); ^{13}C{^{1}H} NMR (75.5 MHz, CD$_3$CN, r.t.): δ = 48.8 (NCH$_2$), 51.1 (NCH$_2$), 69.9 (C$_5$H$_5$), 70.5 (C$_5$H$_4$), 71.4 (C$_5$H$_4$), 76.3 (C$_5$H$_4$), 131.4 (C$_6$H$_4$), 133.0 (C$_6$H$_4$), 139.1 (C$_6$H$_4$), 158.3 (C$_6$H$_4$); elemental analysis: calcd (%) for C$_{18}$H$_{19}$IF$_6$FeNP: C 37.46, H 3.32, N 2.43; found: C 37.65, H 3.33, N 2.40.

*Crystal synthesis of [(FcCH$_2$NH$_2$CH$_2$C$_6$H$_3$-3,4-Cl$_2$)(DB24C8)](PF$_6$) (**1a**)*

Yellow crystals of pseudorotaxane **la** were obtained by slow evaporation of CH$_2$Cl$_2$/Et$_2$O solution of **2a** (52 mg, 0.10 mmol) and DB24C8 (46 mg, 0.10 mmol). **1a** was obtained in 10% yield. Elemental analysis: calcd (%) for C$_{42}$H$_{50}$NO$_8$FeF$_6$Cl$_2$P: C 52.08, H 5.20, N 1.45, Cl 7.32; found: C 51.58, H 4.93, N 1.45, Cl 7.38.

*Crystal synthesis of [(FcCH$_2$NH$_2$CH$_2$C$_6$H$_3$-3,4-F$_2$)(DB24C8)](PF$_6$) (**1b**)*

Yellow crystals of pseudorotaxane **1b** were obtained by slow evaporation of acetone/hexane solution of **2b** (46 mg, 0.093 mmol) and DB24C8 (49 mg, 0.11 mmol).

1b was obtained in 57% yield. Elemental analysis: calcd (%) for $C_{42}H_{50}NO_8FeF_8P$: C 53.91, H 5.39, N 1.50; found: C 53.81, H 5.39, N 1.52.

*Crystal synthesis of ((FcCH$_2$NH$_2$CH$_2$C$_6$H$_4$-4-F)(DB24C8)](PF$_6$) (**1c**)*

Yellow crystals of pseudorotaxane **1c** were obtained by slow evaporation of acetone/hexane solution of **2c** (47 mg, 0.10 mmol) and DB24C8 (44 mg, 0.099 mmol). **1c** was obtained in 49% yield. Elemental analysis: calcd (%) for $C_{42}H_{51}NO_8FeF_7P$: C 54.97, H 5.60, N 1.53; found: C 54.92, H 5.65, N 1.89.

*Crystal synthesis of [(FcCH$_2$NH$_2$CH$_2$C$_6$H$_4$-4-Cl)(DB24C8)](PF$_6$) (**1d**)*

Yellow crystals of pseudorotaxane **1d** were obtained by slow evaporation of acetone/hexane solution of **2d** (48 mg, 0.10 mmol) and DB24C8 (45 mg, 0.10 mmol). **1d** was obtained in 74% yield. Elemental analysis: calcd (%) for $C_{42}H_{51}NClF_6FeO_8P$: C 54.00, H 5.50, N 1.50; found: C 53.69, H 5.45, N 1.48.

*Crystal synthesis of [(FcCH$_2$NH$_2$CH$_2$C$_6$H$_4$-4-Br)(DB24C8)](PF$_6$) (**1e**)*

Yellow crystals of pseudorotaxane **1e** were obtained by slow evaporation of $CHC1_3$/acetone (2.0 mL/0.5 mL) solution of **2e** (53 mg, 0.10 mmol) and DB24C8 (45 mg, 0.10 mmol). **1e** was obtained in 85% yield. Elemental analysis: calcd (%) for $C_{42}H_{51}NBrF_6FeO_8P(H_2O)_{0.5}$: C 51.08, H 5.31, N 1.42; found: C 50.99, H 5.35, N 1.45.

*Crystal synthesis of [(FcCH$_2$NH$_2$CH$_2$C$_6$H$_3$-3-F-4-Me)(DB24C8)](PF$_6$) (**1f**)*

Yellow crystals of pseudorotaxane **1f** were obtained by slow evaporation of CH_2Cl_2/Et_2O solution of **2f** (47 mg, 0.094 mmol) and DB24C8 (45 mg, 0.10 mmol). **1f** was obtained in 41% yield. Elemental analysis: calcd (%) for $C_{43}H_{53}NF_7FeNO_8P$: C 55.43, H 5.73, N 1.50; found: C 55.45, H 5.29, N 1.55.

*Crystal synthesis of [(FcCH$_2$NH$_2$CH$_2$C$_6$H$_4$-4-I)(DB24C8)](PF$_6$) (**1g**)*

Yellow crystals of pseudorotaxane **1g** were obtained by slow evaporation of CH_2C1_2/Et_2O solution of **2g** (57 mg, 0.10 mmol) and DB24C8 (45 mg, 0.10 mmol). **1g** was obtained in 69% yield. Elemental analysis: calcd (%) for $C_{42}H_{51}NIF_6FeNO_8P$: C 49.19, H 5.01, N 1.37; found: C 49.29, H 4.98, N 1.37

Table S1. IR and TGA data of **1a-1g** and **2a-2g**.

compound	IR ν(N-H) /cm^{-1}	$T_{d5}$$^{a)}$/°C
**1a**	3080, 3185	222 (29 $^{c)}$)
**1b**	3066, 3163	222 (23 $^{c)}$)
**1c**	3077, 3165	238 (50 $^{c)}$)
**1d**	3069, 3187	234 (35 $^{c)}$)
**1e**	3069, 3187	231 (36 $^{c)}$)
**1f**	3067, 3166	237 (20 $^{c)}$)
**1g**	3065, 3195	236 (34 $^{c)}$)
**2a**	3233, 3262	193
**2b**	3233, 3262	199
**2c**	3236, 3262	188
**2d**	3233, 3266	199
**2e**	3233, 3265	195
**2f**	3233, 3266	217
**2g**	3235, 3268	202

a) 5% weight loss temperature, b) Not determined, c) T_{d5}(rotaxane) - T_{d5}(axle), rotaxane = **1a-1g**, axle = **2a-2g**

Table S2. Crystallographic results

compound	**1a**	**1b**	**1c**	**1d**
Formula	$C_{42}H_{50}Cl_2F_6FeNO_8P$	$C_{42}H_{50}F_8FeNO_8P$	$C_{42}H_{51}F_7FeNO_8P$	$C_{42}H_{51}ClF_6FeNO_8P$
ccdc	1032569	1032570	1032571	1032572
Temp/K	113	113	113	113
a/Å	10.639(8)	10.418(3)	10.326(2)	10.186(5)
b/Å	10.718(10)	11.204(3)	11.626(2)	11.028(6)
c/Å	19.744(14)	18.120(5)	17.311(3)	19.487(8)
α/°	87.30(3)	89.410(7)	88.365(3)	86.963(15)
β/°	76.15(2))	85.654(7)	86.948(4)	76.898(15)
γ/°	89.12(3)	88.006(6)	88.866(10)	89.125(18)
V/Å^3	2183	2107.5	2072.7	2128.8
crystal system	triclinic	triclinic	triclinic	triclinic
space group	P–1 (No. 2)	P–1 (No. 2)	P–1 (No. 2)	P–1 (No. 2)
Z	2	2	2	2
Mr	968.58	935.65	917.65	941.60
Dx/g cm^{-}3	1.473	1.474	1.444	1.453
μ/mm^{-1}	0.582	0.485	0.488	0.927
F(000)	1004	972	956	972
Nref	9323	9146	8945	9151
R (reflections)	0.1032 (2608)	0.0361 (7353)	0.0346 (6359)	0.0667 (6426)
wR (reflections)	0.1338 (2608)	0.1056 (7353)	0.0791 (6359)	0.2109 (6426)
GOF	0.720	1.011	0.858	1.121
N_{par}	350	568	559	559

No	**1e**	**1f**	**1g**	**1i-Ni**
Formula by ICP	$C_{42}H_{51}BrF_6FeNO_8P$	$C_{43}H_{53}F_7FeNO_8P$	$C_{43}H_{51}F_6FeINO_8P$	$C_{49.5}H_{54}ClFeNNiO_8S_{10}$
	1032573	1032574	1032575	2122024
Temp/K	113	113	113	113
a/Å	10.1826(3)	10.351(3)	10.335(2)	24.469(4)
b/Å	10.9946(5)	10.862(3)	10.991(3)	10.599(2)
c/Å	19.5223(6)	19.572(5)	19.677(4)	21.608(4)
α/°	87.183(5)	87.190(9)	87.097(6)	90
β/°	76.844(5)	76.858(9)	77.503(5)	97.371(2)
γ/°	88.816(6)	89.111(11)	88.510(7)	90
V/$Å^3$	2125.6	2140.4	2144.8	5558.0
crystal system	triclinic	triclinic	triclinic	monoclinic
space group	P–1 (No. 2)	P–1 (No. 2)	P–1 (No. 2)	$P2_1/c$ (No. 14)
Z	2	2	2	2
Mr	978.56	931.68	1025.55	1261.54
Dx/g $cm^{-}3$	1.529	1.446	1.563	1.508
μ/mm^{-1}	1.412	0.474	1.169	1.196
F(000)	1008	972	1044	968
Nref	8877	9280	9346	12361
R (reflections)	0.0375 (7491)	0.0653 (6406)	0.0343 (8084)	0.0596 (10925)
wR (reflections)	0.0798 (7491)	0.1815 (6406)	0.0752 (8084)	0.1123 (10925)
GOF	1.048	1.003	1.017	1.196
N_{par}	549	569	606	667
